# Supplementary material for: Does Acute Caffeine Supplementation Improve Physical Performance in Female Team-Sport Athletes? Evidence from a Systematic Review and Meta-Analysis
Source: Nutrients. 2021 Oct 19;13(10):3663. doi: 10.3390/nu13103663 (PMC8538965; doi:10.3390/nu13103663)
Supplement: Supplementary file 1 [file nutrients-13-03663-s001.zip › nutrients-1392790-supplementary.pdf]

Supplementary Table S1. Search strategy

	Pubmed	SportDiscuss	SCOPUS
Caffeine keywords	(((((“caffeine”[MeSH Terms] OR “caffeine”[All Fields]) OR (“energy drinks”[MeSH Terms] OR (“energy”[All Fields] AND “drinks”[All Fields]) OR “energy drinks”[All Fields] OR (“energy”[All Fields] AND “drink”[All Fields]) OR “energy drink”[All Fields])) OR caffeinated[All Fields]) OR (“tea”[MeSH Terms] OR “tea”[All Fields])) OR (“coffee”[MeSH Terms] OR “coffee”[All Fields]))	(DE "ENERGY drinks") OR "Energy drinks" OR (DE "CAFFEINE") OR "Caffeine drinks" OR "Caffeinated" OR "Coffee"	“caffeine” OR “energy drinks” OR (“energy” AND “drink”) OR “energy drink” OR “caffeinated” OR “tea” OR “coffee”
	AND		
Team sports keywords	((((((((((((((team[All Fields] AND (“sports”[MeSH Terms] OR “sports”[All Fields] OR “sport”[All Fields])) OR (“volleyball”[MeSH Terms] OR “volleyball”[All Fields])) OR (“soccer”[MeSH Terms] OR “soccer”[All Fields])) OR (“football”[MeSH Terms] OR “football”[All Fields])) OR (“basketball”[MeSH Terms] OR “basketball”[All Fields])) OR (“hockey”[MeSH Terms] OR “hockey”[All Fields])) OR handball[All Fields]) OR croquet [All Fields]) OR netball[All Fields]) OR “water polo”[All Fields]) OR waterpolo[All Fields]) OR futsal[All Fields]) OR (“football”[MeSH Terms] OR “football”[All Fields] OR “rugby”[All Fields])) OR (“baseball”[MeSH Terms] OR “baseball”[All Fields])) OR floorball[All Fields]) OR (“baseball”[MeSH Terms] OR “baseball”[All Fields] OR “softball”[All Fields])).	(DE "TEAM sports") OR "TEAM SPORTS" OR (DE "VOLLEYBALL") OR "Volleyball" OR (DE "SOCCER") OR "soccer" OR (DE "FOOTBALL") OR "Football" OR (DE "BASKETBALL") OR "Basketball" OR (DE "HOCKEY") OR "Hockey" OR (DE "HANDBALL") OR "Handball" OR (DE "NETBALL") OR "Netball" OR (DE "WATER polo") OR Waterpolo OR "Water polo" OR (DE "INDOOR soccer") OR "Futsal" OR (DE "RUGBY football") OR "Rugby" OR (DE "BASEBALL") OR "Baseball" OR (DE "INDOOR hockey") OR "floorball" OR (DE "SOFTBALL") OR "Softball"	(“team” AND “sports”) OR “team sport” OR “team sports” OR “volleyball” OR “soccer” OR “football” OR “basketball” OR “hockey” OR “handball” OR “netball” OR “water polo” OR “waterpolo” OR “futsal” OR “rugby” OR “baseball” OR “floorball” OR “softball”
**Results**	**217 documents**	**290 documents**<br>**Filter:**<br>**“Peer reviewed articles”**<br>**115 documents**	**328 documents**<br>**Filter: Document type: “Article”:**<br>**256 documents**

Supplementary Table S2. Quality Assessment of included randomized controlled trials (PEDro scores)

Author/year	Random allocation	Concealed allocation	Similarity at baseline	Subject blinding	Therapist blinding	Assessor blinding	>85% follow-up for ≥1 primary outcome	All participants or ITT analysis	Between-group statistical comparison for ≥1 key outcome	Point measures and variability	PEDro score
Astorino TA. et al. 2011	1	1	1	1	0	0	1	1	1	1	8/10
Del Coso J. et al. 2013	1	1	1	1	1	1	1	1	1	1	10/10
Lee CL. et al. 2014	1	1	1	1	1	1	1	1	1	1	10/10
Lara B. et al. 2014	1	1	1	1	1	1	1	1	1	1	10/10
Buck C. et al. 2015	1	1	1	1	1	1	1	1	1	1	10/10
Chen HY. et al. 2015	1	1	1	1	1	1	1	1	1	1	10/10
Mahdavi R. et al. 2015	1	1	1	1	1	1	1	1	1	1	10/10
Fernández-Campos C. et al. 2015	1	1	1	1	1	1	1	0	1	1	9/10
Pérez-López A. et al. 2015	1	1	1	1	1	1	1	1	1	1	10/10
Ali A. et al. 2016	1	1	1	1	1	1	1	1	1	1	10/10
Ali A. et al. 2016	1	1	1	1	1	1	1	1	1	1	10/10
Portillo J. et al. 2017	1	1	1	1	1	1	1	1	1	1	10/10
Puente C. et al. 2017	1	1	1	1	1	1	1	1	1	1	10/10
Pfeifer DR. et al. 2017	1	1	1	1	0	0	1	1	1	1	8/10
Stojanovic E, et al. 2019	1	1	1	1	1	1	1	1	1	1	10/10
Tan ZS. et al. 2020	1	1	1	1	0	0	1	1	1	1	8/10
Muñoz A. et al. 2020	1	1	1	1	1	1	1	1	1	1	10/10
Karayigit R. et al. 2021	1	1	1	1	1	1	1	1	1	1	10/10

ITT: intention to treat; PEDro: Physioterapy Evidence Database.
Tool used: PEDro scale
Each item has been scored as 1 when it is presented and as 0 when it is absented. PEDro score is obtained after summing all items.

Supplementary Table S3. Quality Assessment of included randomized controlled trials (RoB 2 tool)

Study	Risk of bias domains					
	D1	D2	D3	D4	D5	Overall
Astorino TA. et al. 2011	+	-	+	-	+	-
Del Coso J. et al. 2013	+	+	+	+	+	+
Lee CL. et al. 2014	+	+	+	+	+	+
Lara B. et al. 2014	+	+	+	+	+	+
Buck C. et al. 2015	+	+	+	+	+	+
Chen HY. et al. 2015	+	+	+	+	+	+
Mahdavi R. et al. 2015	+	+	+	+	+	+
Fernández-Campos C. et al. 2015	+	-	+	+	+	-
Pérez-López A. et al. 2015	+	+	+	+	+	+
Ali A. et al. 2016a	+	+	+	+	+	+
Ali A. et al. 2016b	+	+	+	+	+	+
Portillo J. et al. 2017	+	+	+	+	+	+
Puente C. et al. 2017	+	+	+	+	+	+
Pfeifer DR. et al. 2017	+	-	+	-	+	-
Stojanovic E, et al. 2019	+	+	+	+	+	+
Tan ZS. et al. 2020	+	-	+	-	+	-
Muñoz A. et al. 2020	+	+	+	+	+	+
Karayigit R. et al. 2021	+	+	+	+	+	+

Domains:
D1: Bias arising from the randomization process.
D2: Bias due to deviations from intended intervention.
D3: Bias due to missing outcome data.
D4: Bias in measurement of the outcome.
D5: Bias in selection of the reported result.

Judgement
- Some concerns
+ Low
